# Supplementary material for: Correlation between Psychosomatic Assessment, Heart Rate Variability, and Refractory GERD: A Prospective Study in Patients with Acid Reflux Esophagitis
Source: Life (Basel). 2023 Sep 3;13(9):1862. doi: 10.3390/life13091862 (PMC10533115; doi:10.3390/life13091862)
Supplement: Supplementary file 1 [file life-13-01862-s001.zip › life-2472361-supplementary.pdf]

Supplementary Table S1. Heart rate variability (HRV) parameters.

HRV Parameter (Unit)	Description (normal range)	Represents
HR (Heart Rate) (per min)	Number of heartbeats per minute (60-100)	Higher heart rate may indicate sympathetic dominance, while lower heart rate may indicate parasympathetic dominance
SD (Spectral Distribution)	Analysis of frequency components in HRV	The distribution of power in different frequency bands
RRIV (R-R Interval Variation) (ms)	Variation in duration between successive R waves (19–75)	Higher RRIV reflects greater HRV and better autonomic function
TP (Total Power) (ms^2)	Total power in HRV spectrum	Overall variability and autonomic function
VL (Very Low Frequency) (Hz)	Power in the very low-frequency range (0.003–0.04 Hz)	Sympathetic modulation and slower physiological processes
LF (Low Frequency) (ms^2)	Power in the low-frequency range (0.04 to 0.15 Hz) (193–1,009)	A mixture of sympathetic and parasympathetic influences
HF (High Frequency) (ms^2)	Power in the high-frequency range (0.15 to 0.4 Hz) (83–3,630)	Parasympathetic (vagal) modulation of heart rate
NN (Normal-to-Normal Interval)	Interval between consecutive normal heartbeats (785–1,160)	Calculate various HRV parameters
HRV_ANS age (y/o)	HRV parameters adjusted for age-related changes	Provides age-specific assessment of HRV
ANS (Autonomic Nervous System) Balance	Balance between sympathetic and parasympathetic activity	A more balanced ANS activity is associated with better cardiovascular health
SDNN (Standard Deviation of Normal-to-Normal) (ms)	Standard deviation of NN intervals (32–93 ms)	Overall HRV and autonomic regulation.
In (LF/HF) (Low Frequency/High Frequency)	Ratio of low-frequency power to high-frequency power	Sympathovagal balance and autonomic modulation

Abbreviations: HRV, heart rate variability.

Supplementary Table S2. Variations between before and after treatments for symptoms score.

Variate/Time	W0	W4	W8	W16	W24	W48	p-value
GERDQ	9.0±2.4*	7.6±2.0*	6.7±1.7*	7.4±1.4	7.2±1.3	6.9±1.4	*<0.001
GERDQLQ	25.3±17.2	13.8±12.7	7.8±11.3				<0.001
RSI	15.1±8.5	8.8±6.8	5.9±5.6				<0.001
BAI	6.6±6.6	4.2±4.9	3.2±4.4				<0.001
BDI	7.1±6.7	4.7±5.2	3.9±5.0				<0.001
SSD-8	10.9±6.8	7.0±5.9	4.9±5.3				<0.001

Abbreviations: GERDQ, GERD-questionnaire; GERDQLQ, GERD-Quality of Life Questionnaire; RSI, reflux symptom index; BAI, Beck anxiety inventory; BDI, Beck depression inventory; SSD-8, 8-item somatic symptom scale.
